# Systematics of a Kleptoplastidal Dinoflagellate, *Gymnodinium eucyaneum* Hu (Dinophyceae), and Its Cryptomonad Endosymbiont

**DOI:** 10.1371/journal.pone.0053820

**Published:** 2013-01-07

**Authors:** Shuang Xia, Qi Zhang, Huan Zhu, Yingyin Cheng, Guoxiang Liu, Zhengyu Hu

**Affiliations:** 1 Key Laboratory of Algal Biology, Institute of Hydrobiology, Chinese Academy of Sciences, Wuhan, People's Republic of China; 2 Graduate school of Chinese Academy of Sciences, Beijing, People's Republic of China; 3 Center for Water Envionment and Human Health, Institute of Hydrobiology, Chinese Academy of Sciences, Wuhan, People's Republic of China; Uppsala University, Sweden

## Abstract

New specimens of the kleptoplastidal dinoflagellate *Gymnodinium eucyaneum* Hu were collected in China. We investigated the systematics of the dinoflagellate and the origin of its endosymbiont based on light morphology and phylogenetic analyses using multiple DNA sequences. Cells were dorsoventrally flattened with a sharply acute hypocone and a hemispherical epicone. The confusion between *G. eucyaneum* and *G. acidotum* Nygaard still needs to be resolved. We found that the hypocone was conspicuously larger than the epicone in most *G. eucyaneum* cells, which differed from *G. acidotum*, but there were a few cells whose hypocone and epicone were of nearly the same size. In addition, there was only one site difference in the partial nuclear LSU rDNA sequences of a sample from Japan given the name *G. acidotum* and *G. eucyaneum* in the present study, which suggest that *G. eucyaneum* may be a synonym of *G. acidotum*. Spectroscopic analyses and phylogenetic analyses based on nucleomorph SSU rDNA sequences and chloroplast 23 s rDNA sequences suggested that the endosymbiont of *G. eucyaneum* was derived from *Chroomonas* (Cryptophyta), and that it was most closely related to *C. coerulea* Skuja. Moreover, the newly reported kleptoplastidal dinoflagellates *G. myriopyrenoides* and *G. eucyaneum* in our study were very similar, and the taxonomy of kleptoplastidal dinoflagellates was discussed.

## Introduction

Dinoflagellates are a diverse group of single-celled eukaryotic algae that occur in marine and freshwater all over the world [1]. Some have acquired chloroplasts via endosymbiosis [2]. This phenomenon provides insights into the Serial Endosymbiosis Theory that some algal groups arose via the ingestion and retention of photosynthetic, eukaryotic organisms and the subsequent reduction of their nonphotosynthetic organelles [3], [4], [5]. The origins and structures of endosymbionts are highly diverse. *Karenia* Hansen & Moestrup, *Karlodinium* Larsen, and *Takayama* de Salas have chloroplasts that originated from haptophyte algae, which are surrounded by three membranes but no other organelles remain from the endosymbiont [6], [7]. *Durinskia* Carty & Cox, *Kryptoperidinium* Lindemann, and *Peridinium* Ehrenberg contain chloroplasts derived from diatoms [8], [9]. The nucleus and mitochondria of the diatom remain Dinophyceae in the host cell where they are surrounded by a single membrane [10], [11]. The chloroplasts of *Dinophysis* Ehrenberg originated from a cryptophyte, probably *Teleaulax* Hill [12], [13], [14], [15], and they are surrounded by two membranes [16].

The retention time of plastids in dinoflagellates also varies greatly depending on the species involved and the conditions under which they are grown [17]. Some endosymbionts are permanent, whereas others are engulfed and temporarily retained in a functional state for a few weeks. The temporary retention of engulfed chloroplasts is known as “kleptoplastidy” and the endosymbionts (chloroplasts) are referred to as “kleptochloroplasts” [18]. The relationship between the endosymbiont and the host remains obscure. In a recent study of *Dinophysis acuminata* Claparède & Lachmann, it was observed that the kleptoplastids were serviced by nucleus-encoded proteins and horizontal gene transfer from the endosymbiont to the host nucleus was detected [19]. Therefore, studies of kleptoplastidy are very interesting and important for increasing our understanding of endosymbiosis and the evolution of algae.

A relatively small group of dinoflagellates have been described as having a blue-green coloration and researchers are keen to understand the source of their coloration [20]. Studies of *Gymnodinum acidotum* Nygaard, *G. aeruginosum* Stein, and *G. myriopyrenoides* Yamaguchi, Nakayama, Kai et Inouye had indicated that a cryptophycean endosymbiont was housed temporarily within the dinophycean cell, which was the source of the blue-green chloroplasts [20], [21], [22], [23], [24]. The discovery of nonphotosynthetic organelles in the endosymbionts in dinophycean cells suggested that these are examples of an early stage in the evolutionary process [23]. Thus, systematic studies of this group may be of great evolutionary interest. However, most previous studies are based on pigmentation and morphological observation, whereas the phylogenetic relationships among the blue-green group of dinoflagellates and their endosymbionts remain uncertain.

The blue-green freshwater dinoflagellate *Gymnodinium eucyaneum* Hu (Hu et al. 1980, as *G. cyaneum*; Hu 1983) was originally described from China as processing phycobilin like cryptomonads, suggesting that it probably contained a cryptophycean endosymbiont [25], [26]. At present, it has only been reported in China. In this study, we collected new specimens of *G. eucyaneum* from China and their cell morphology was observed by light microscopy, while the systematics of the dinoflagellates were investigated via phylogenetic analyses based on partial nuclear LSU rDNA sequences. To identify the origin of the endosymbiont, the nucleomorph SSU rDNA and chloroplast 23S rDNA sequences were determined, and the absorption spectrum of the phycocyanin was measured. The sequences of some cryptomonads were also determined for reference.

## Results

### Description

*Gymnodinium eucyaneum* (Hu, Yu et Zhang) Hu 1983, Hu, p.198–199; *Gymnodinium cyaneum* Hu, Yu et Zhang 1980, Hu et al., p. 651–653. Non *Gymnodinium cyaneum* Schiller 1955.

**Figure 1:**
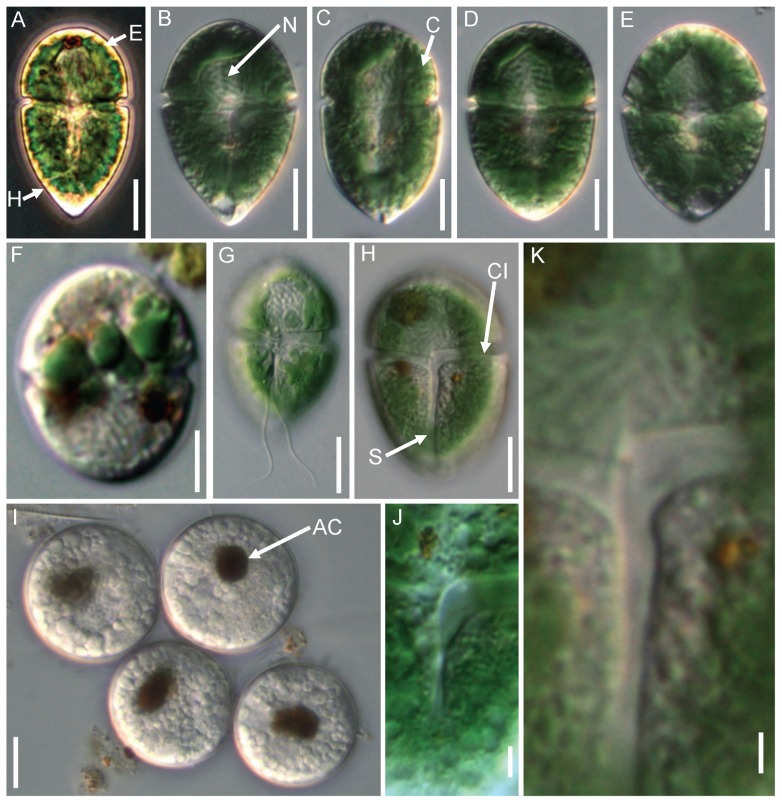
Micrographs of *Gymnodinium eucyaneum*. **Figs. A–E.** Different cell shapes of the field samples. **F** Cells kept for 2∼4 weeks in the laboratory, showing that the chloroplast became smaller. **G** Ventral view showing the insertion of the flagella. **H, J, K** Ventral view showing the detail of the cingulum and sulcus. **I** Cysts each with a brownish accumulation of corpuscles. E: epicone; H: hypocone; N: nucleus; C: chloroplasts; CI: cingulum; S: sulcus; AC: accumulation of corpuscle. Scale bars: A–I = 10 μm; J–K = 2 μm.

Unicellular, freshwater unarmored dinoflagellate. Cells were obviously dorsoventrally flattened, measuring 29∼48 μm in length, 16∼22 μm in width, and 12∼17 μm in thickness. In most cases, the hypocone was conspicuously larger than the epicone (Figs. 1A–D). The epicone was hemispherical and its length was approximately one-third of the total cell length (Figs. 1A–D). The hypocone was sharply acute (Figs. 1A–E). In a few cells, the hypocone and epicone were more or less the same size (Fig. 1E). The cingulum was wide, deeply excavated, and encircled the middle-upper part of the cell (Figs. 1G, H, J, K). There was no displacement of the cingulum and its ventral ends were at the same level, where both curved posteriorly at the junction with the sulcus (Figs. 1G, H, J, K). The sulcus was wide, expanding into the posterior part (Figs. 1G, H, J, K). Two flagella were inserted on the ventral side of the cingulum (Fig. 1G).

A large spherical nucleus was situated in the anterior part of cell (Figs. 1B–E, G, H). Hundreds of granules of variable size were observed beneath the plasmalemma (Fig. 1). Numerous blue-green chloroplasts were located peripherally in the cell (Figs. 1A–H). Determining the actual number was difficult because they were very dense. The chloroplasts gradually became smaller when cells were retained in the lab (Fig. 1F). Colorless cysts with a brownish accumulation of corpuscle formed after 2∼4 weeks culture in filtered local water (Fig. 1I).

The voucher specimens examined were: HBI 3586× from Lake Donghu in Wuhan City, Hubei Province, collected by SX on April 18, 2012; HBI 3597× from a fishpond in Wuhan City, Hubei Province, collected by SX on May 3, 2012. The specimens are deposited in the Freshwater Algal Herbarium (HBI), Institute of Hydrobiology, Chinese Academy of Sciences, Wuhan, Hubei, China.

### Spectroscopy

**Figure 2:**
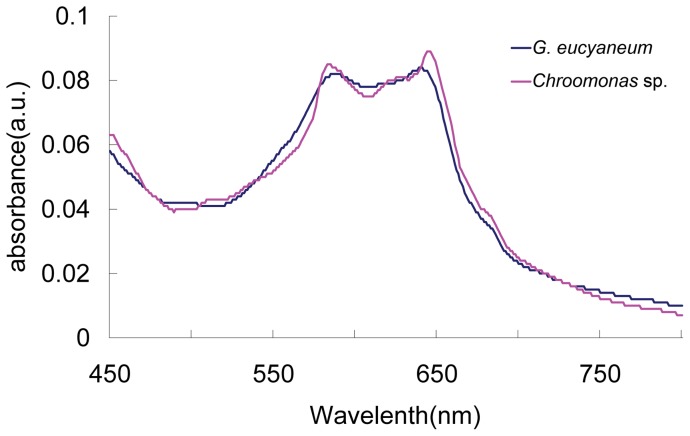
Absorption spectrum of phycocyanin extracted from *Gymnodinium eucyaneum* samples and a *Chroomonas* sp. strain.

The absorption spectrum of the phycocyanin extracted from *G. eucyaneum* in this study is shown in Fig. 2. Two absorption peaks were observed at 641 nm and 585 nm. The peak at 641 nm was slightly higher than the peak at 585 nm.

### Phylogenetic Analyses

#### Host phylogeny based on the nuclear LSU rDNA

**Figure 3:**
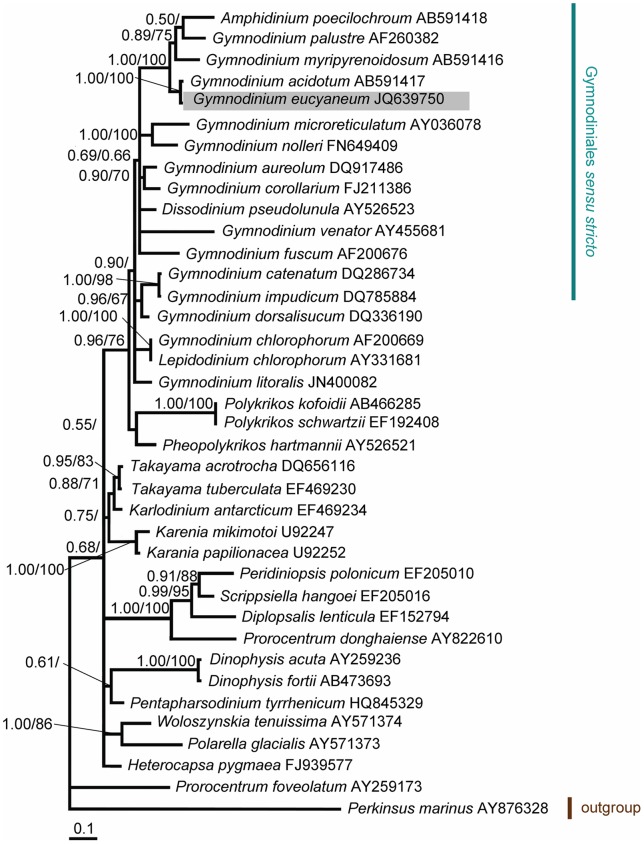
Bayesian phylogenetic tree constructed from the nuclear LSU rDNA sequences. The numbers on the nodes represent the posterior probabilities (PP)/bootstrap support values (BP) produced by the Bayesian inference and maximum-likelihood analyses. Values >0.50 for PP and >50 for BP are shown. The sequences obtained in our study are shaded in gray.

The LSU rDNA sequences aligned in this study contained 572 nucleotides for 38 taxa of dinoflagellates and their putative relatives. Of these nucleotides, 389 sites (68.0%) were variable and 309 sites (54.0%) were parsimoniously informative. The base frequencies were found to be homogeneous across taxa. The overall average pairwise distance was 0.253. The phylogenetic trees constructed by the ML and Bayesian analyses produced similar topologies to their composition, although only the Bayesian trees are presented. In the phylogenetic tree, the members of the genus *Gymnodinium* sensu stricto formed a well supported clade (0.96/76 for BA/ML) (Fig. 3). *G. eucyaneum* was present in this clade and it formed a robust subclade (1.00/100 for BA/ML) with *G. acidotum*. The interspecific pairwise divergence between them was 0.002 and there was only one site difference. Both clustered with *G. palustre, G. myripyrenoidosum,* and *Amphidinium poecilochroum* with high support (1.00/100 for BA/ML). The interspecific pairwise divergence between *G. eucyaneum* and *G. myripyrenoidosum* was 0.108. This group did not show strong affinity to any others.

### Endosymbiont phylogeny based on the nucleomorph SSU rDNA and chloroplast 23S rDNA

**Figure 4:**
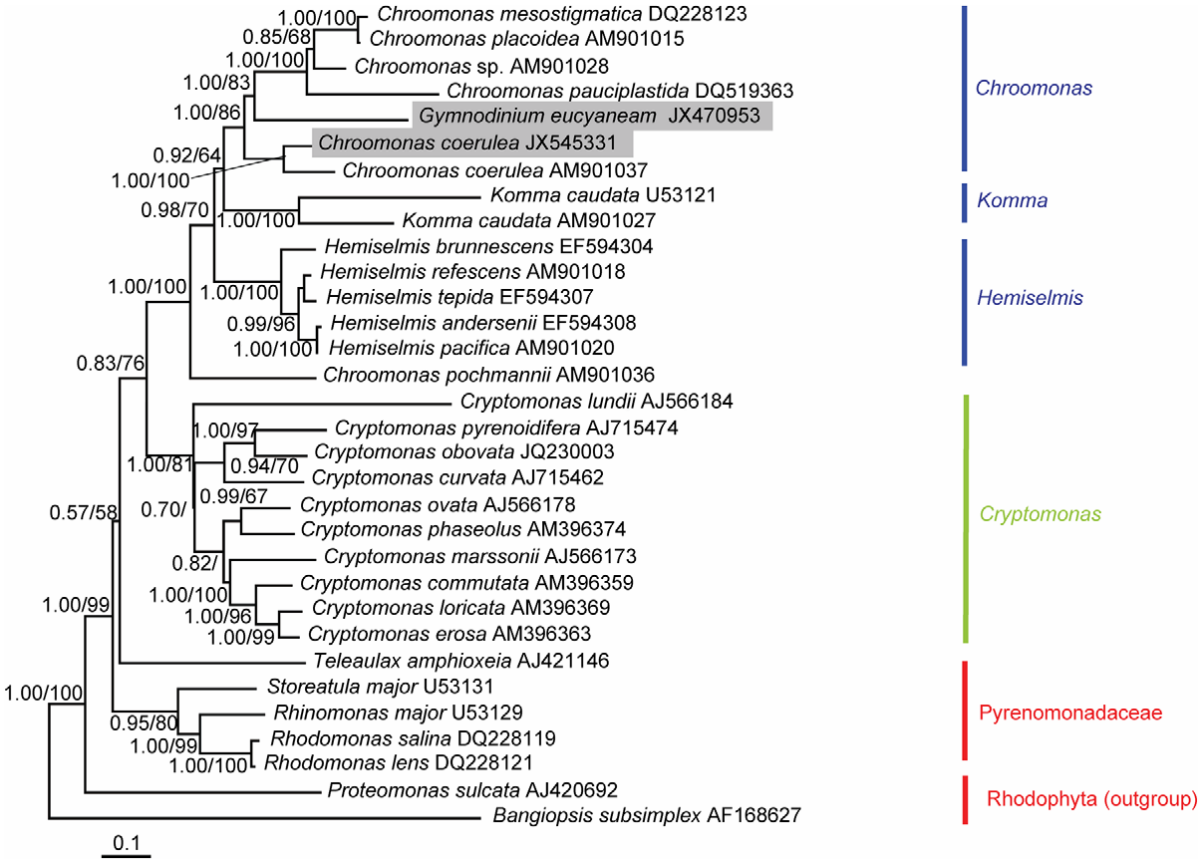
Bayesian phylogenetic tree constructed from the nucleomorph SSU rDNA sequences. The numbers on the nodes represent the posterior probabilities (PP)/bootstrap support values (BP) produced by the Bayesian inference and maximum-likelihood analyses. Values >0.50 for PP and >50 for BP are shown. The sequences obtained in our study are shaded in gray.

The nucleomorph SSU rDNA sequences aligned in this study contained 1872 nucleotides for 32 taxa of dinoflagellates, cryptomonads, and their putative relatives. Of these nucleotides, 741 sites (39.6%) were variable and 477 sites (25.5%) were parsimoniously informative. The base frequencies were found to be homogeneous across taxa. The overall average pairwise distance was 0.077. Three main clades were distinguished in the phylogenetic tree, which represented the blue-green, brownish-green, and red cryptomonad species (Fig. 4). The sequence of *G. eucyaneum* was positioned in the clade containing *Chroomonas* sp., *C. mesostigmatica, C. placoidea, C. pauciplastida,* and *C. coerulea* with high support (1.00/86 for BA/ML). The sequence of *G. eucyaneum* was most closely related to two *C. coerulea* sequences. One was from *C. coerulea* strain UTEX 2780 and the other was from *C. coerulea* collected from the lake where *G. eucyaneum* was collected in the present study. Another member of *Chroomonas, C. pochmannii*, was distantly related to this group. The *Komma* and *Hemiselmis* species, which are also blue-green in color, formed two robust groups (1.00/100 for BA/ML and 1.00/100 for BA/ML) with relatively distant relationships to *G. eucyaneum*.

**Figure 5:**
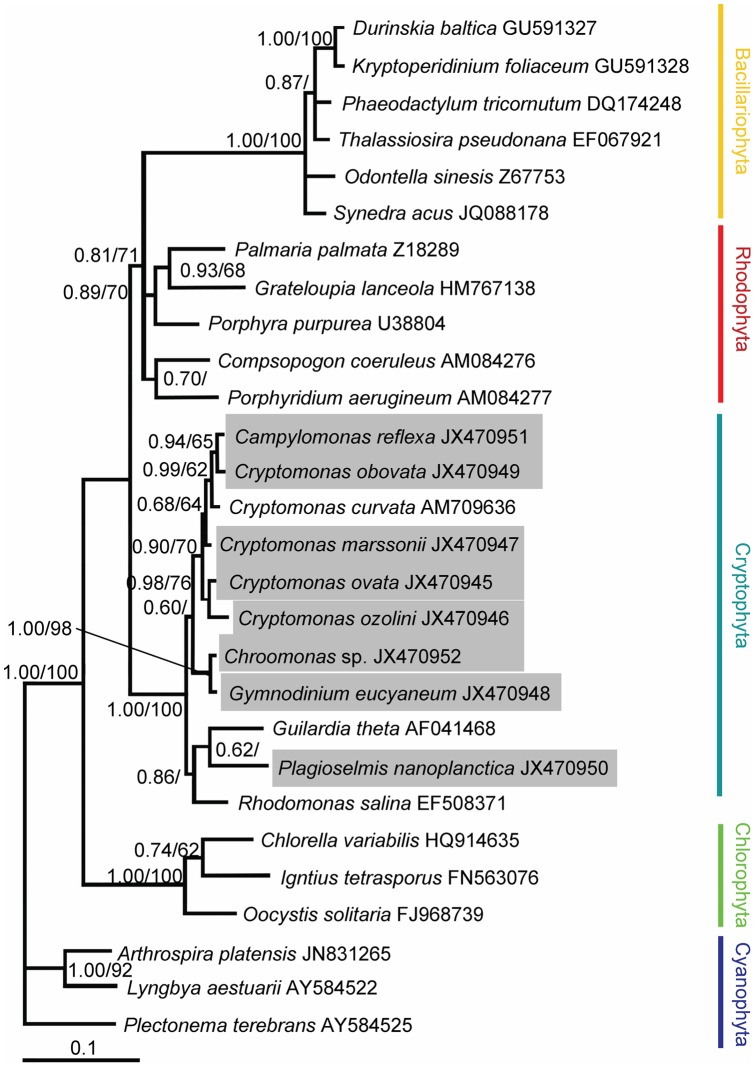
Bayesian phylogenetic tree constructed from the chloroplast 23S rDNA sequences. The numbers on the nodes represent the posterior probabilities (PP)/bootstrap support values (BP) produce by the Bayesian inference and maximum-likelihood analyses. Values >0.50 for PP and >50 for BP are shown. The sequences obtained in our study are shaded in gray.

The chloroplast 23S rDNA sequences aligned in this study contained 976 nucleotides for 28 taxa of cryptomonads, dinoflagellates, diatoms, and other algae. Of these nucleotides, 292 sites (29.9%) were variable and 523 sites (22.2%) were parsimoniously informative. The base frequencies were found to be homogeneous across taxa. The overall average pairwise distance was 0.090. The algae from different phyla were well separated in the reconstructed phylogenetic tree (Fig. 5). The sequence of *G. eucyaneum* was positioned in the cryptomonad clade with high support (1.00/100 for BA/ML). The sequences of *G. eucyaneum* and *C. coerulea* from the same lake formed a robust lineage (1.00/98 for BA/ML) and the pairwise distance between them was 0.001. Several diatoms and dinoflagellates that contained endosymbiont derived from diatoms formed a well-supported clade (1.00/100 for BA/ML).

## Discussion

### Previous studies of *Gymnodinium eucyaneum* in China

Several studies have investigated *G. eucyaneum* in China since 1980 [25]–[29]. However, they are not widely known because they were written in Chinese. Evidence for the presence of phycobilin [25] and the ultrastructure of the chloroplasts [29] suggested that the chloroplasts of *G. eucyaneum* were derived from cryptophytes. Observations of the nucleus and nuclear substance [27] showed that one dinokaryon was present in all cells and the numbers of second eukaryotic nuclei ranged from 0–4 (rarely 7–10). This data may suggest that the second eukaryotic nuclei were temporary and that the chloroplasts of *G. eucyaneum* were “stolen” and could be lost. Similar report on the number of nuclei was made by Field and Rhodes [22].

### *Gymnodinium eucyaneum* and *G. acidotum*

**Figure 6:**
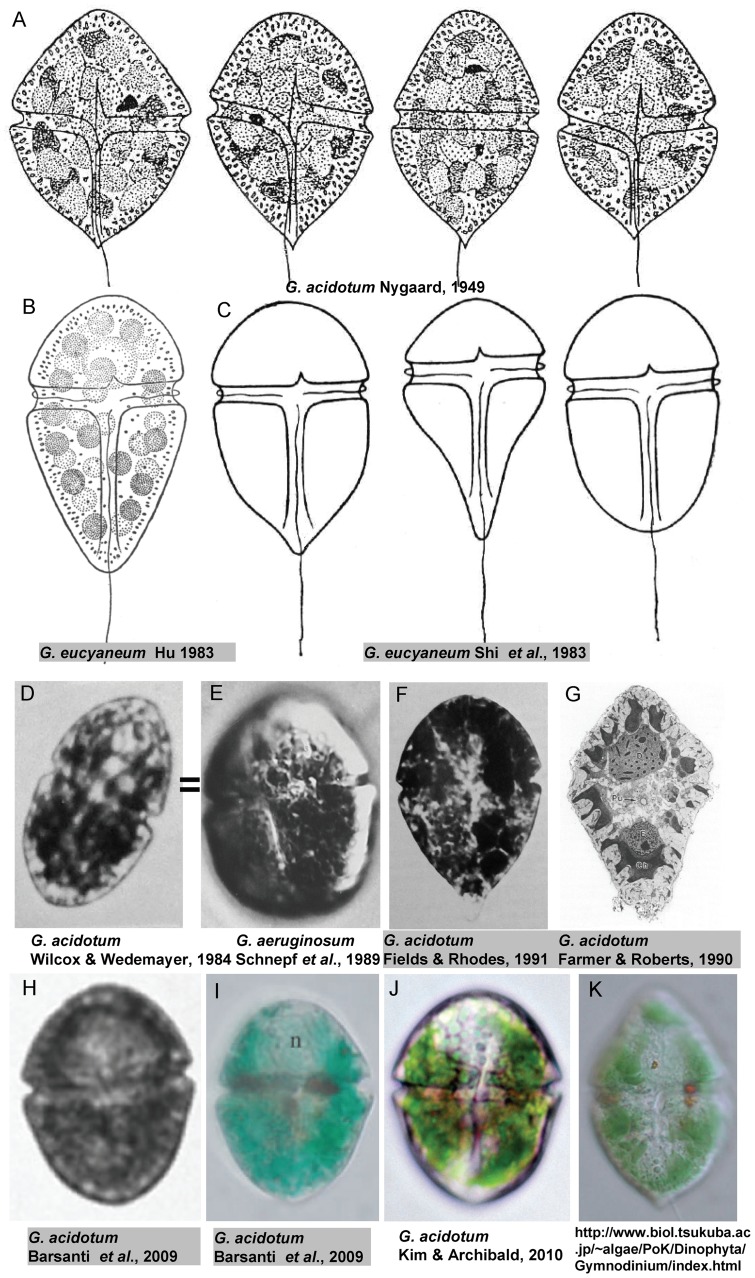
Drawings and photographs of *Gymnodinium eucyaneum* and *G. acidotum*. The organisms shown in F–I were considered to be the same as *G. eucyaneum* in the present study.

Since it was first described in China, *G. eucyaneum* has often been confused with another blue-green unarmored dinoflagellate, *G. acidotum*. Traditionally, unarmored dinoflagellates have been classified based mainly on the relative sizes of the epicone and hypocone [30]. According to their original descriptions, the epicone and the hypocone were nearly equal in *G. acidotum* [31] (Fig. 6A), whereas the hypocone was conspicuously larger than the epicone in *G. eucyaneum* [26], [27] (Figs. 6B, 6C). The hypocone was 1.3∼1.8 times as long as the epicone in *G. eucyaneum* according to our observations. Schnepf et al. (1989) suspected that the organism studied by Wedemayer (1984) under the name “*Gymnodinium acidotum*” was identical to *G. aeruginosum* [24] (Figs. 6D–E). After examining the relative sizes of the epicone and hypocone in the images provided in previous studies, we considered that the organisms studied by Farmer and Roberts (1990) [21], Fields and Rhodes (1991) [22], and Barsanti et al. (2009) [32] (Figs. 6F, G, H, I) under the name “*G. acidotum*” were different from the lectotype and *G. acidotum* in other studies (Figs. 2A, J, K), and they may be identical with the *G. eucyaneum* analyzed in our study. However, recent studies indicate that the classification based on the relative sizes of the epicone and hypocone does not reflect their phylogenetic relationships [7], [33], [34], [35]. In a recent study by Yamaguchi et al. (2011) [23], a partial nuclear LSU rDNA sequence of *G. acidotum* were included. There was only one site difference in the nuclear LSU rDNA sequence of *G. acidotum* in that study and *G. eucyaneum* in the present study. An image of *G. acidotum* was not provided in that paper, but the authors gave us the usage of a photo of *G. acidotum* collected from the same sample on their web site. (Fig. 6K) In the photo, the hypocone and the epicone were more or less the same size. While in our samples of *G. eucyaneum*, the epicone and hypocone were more or less the same size in some cells, although cells of this type were rare. We thought the relative size of epicone and hypocone may varies in different phases of lifecycle or in different habitats. Therefore, *G. eucyaneum* may be a synonym of *G. acidotum.* However, we prefer to postpone the synonymization until more morphological and molecular information on these organisms becomes available.

### *Gymnodinium eucyaneum* and other kleptoplastidal dinoflagellates

Recently, a new unarmored blue-green kleptoplastidal dinoflagellate, *Gymnodinium myriopyrenoides* Yamaguchi, Nakayama, Kai et Inouye was reported from Isonoura Beach in Japan [23]. Although the habitat of *G. myriopyrenoides* was marine and sand-dwelling, while *G. eucyaneum* was a freshwater species. we thought *G. myriopyrenoides* and *G. eucyaneum* were quite similar: in our phylogenetic analyses, the two were very close to each other; as to morphological characters, both of the two were dorsoventrally flattened and elongate-elliptical in ventral view; their epicone was conspicuously smaller than the hypocone; and they both had a wide, deeply incised cingulum with no displacement; both their endosymbionts came from blue-green cryptomonads. The symbiont of *G. myriopyrenoides* was found to be derived from *Chroomonas* or *Hemiselmis* via phylogenetic analyses based on plastid-encoded SSU rDNA, but it could not be identified at species level because plastid-encoded SSU rDNA sequeces of *Chroomonas* and *Hemiselmis* were insufficient. In the present research, the symbiont of *G. eucyaneum* was confirmed to be derived from *Chroomonas* via phylogenetic analyses based on nucleomorph SSU rDNA and spectrophotometric pigment analyses.

*G. myriopyrenoides* and *G. eucyaneum*, together with some other species of *Gymnodinium* and *Amphidinium* who also harbored blue-green kleptochloroplasts, such as *G. aeruginosum, A. poecilochroum, A. latum* and *A. wigrense*, formed a relatively distinct group in Dinophyceae in view of their kleptoplastidal behavior, morphological characters and close relationships in phylogenetic analyses. As mentioned above, recent ultrastructural and molecular phylogenetic studies revealed that the traditional taxonomy of unarmored dinoflagellates based mainly on the relative sizes of the epicone and hypocone was problematic. As revealed in ultrastructural and molecular phylogenetic studies, the genus *Gymnodinium* sensu Hansen er Moestrup and *Amphidinium* were polyphyletic [33], [34], [35], [36]. Yamaguchi et proposed to establish a new genus for these kleptoplastidal dinoflagellates based on morphological and molecular characters, and we considered this proposal was more reasonable than the traditional taxonomy.

### Identification of the endosymbiont

Previous studies based on spectrophotometric pigment analyses suggested that the phycocyanins in *G. eucyaneum* resembled PC 645 and that *G. eucyaneum* may contain a blue-green cryptomonad endosymbiont [25], although the origin of the endosymbiont remained uncertain. Three cryptomonad genera contain blue-green chloroplasts [37], i.e., *Hemiselmis, Komma*, and *Chroomonas*, and *Komma* and *Chroomonas* both contain PC 645 [38]. The absorption spectrum of the phycocyanin extracted from *G. eucyaneum* in this study was almost the same as that extracted from *G. eucyaneum* in a previous study [25], which matched the PC 645 extracted from *Chroomonas* sp. strain CCMP 1221 [38]. In the phylogenetic analyses based on the nucleomorph SSU rDNA and chloroplast 23S rDNA in this investigation, the sequences of the endosymbiont were firmly included in the *Chroomonas* clade and they had relatively long distances from *Komma* and *Hemiselmis*. Thus, we suggest that the endosymbiont originated from *Chroomonas*. Furthermore, the sequence of the endosymbiont indicated that it was most closely related to two *C. coerulea* sequences. It is quite remarkable that *C. coerulea* and *G. eucyaneum* were collected from the same lake in the same month, in the present study. Thus, we suspect that the endosymbiont of *G. eucyaneum* detected in this study originated from *C. coerulea*. However, the species level identification could be problematic because the taxon sampling and taxonomic studies of *Chroomonas* were inadequate. In addition, DNA changes may have occurred after the endosymbiont was engulfed by the host, which may make the species level identification more complex.

In our study, *G. eucyaneum* survived for 2∼4 weeks without feeding, so the chloroplast was likely to be the nutritional source for the dinoflagellate host, which agreed with previous observations of starch grain accumulation in some kleptoplastidal dinoflagellates [39], [40]. The chloroplasts gradually became smaller and fewer, and host cell division was rarely seen, while attempts to establish clonal cultures failed. In a previous Chinese study, it was reported that the location and number of the nuclear substances (probably cryptomonad nuclei and nucleomorphs) varied greatly among the *G. eucyaneum* cells in the same samples, because the nuclear substances were randomly distributed to two daughter cells when host cell division occurred [27]. These studies suggest that the endosymbiont of *G. eucyaneum* is neither a food nor a permanent endosymbiont and that *G. eucyaneum* is a kleptoplastidal dinoflagellate.

### Why cryptomonads?

In some dinoflagellates, the symbionts are derived from *Chroomonas* and other cryptomonads, such as *Teleaulax*. spp and *Rhodomonas* spp. [39], [41], [42], [43]. Thus, these cryptomonads must have unique characteristics that allow them to become symbionts of dinoflagellates. We propose four main reasons for this phenomenon, as follows.

First, cryptomonads are an appropriate size for engulfment by dinoflagellates.

Furthermore, the cell surface of cryptomonads is a delicate proteinaceous periplast rather than a cellulose wall, which is found only in cryptomonads, although the same term is applied to euglenoids but they have a different arrangement [44]. The periplast is vulnerable to rupturing or distortion [45], so it is probably easily disrupted and digested by the hosts after engulfment.

Moreover, the periplastidial compartment (periplastidial complex or chloroplast endoplasmic reticulum) may maintain a relatively appropriate intracellular environment for the endosymbiont in the host cells.

Finally, the chloroplast of cryptomonads may have the ability to adapt to new intracellular environments. It is widely believed that cryptomonads obtain their chloroplasts by ingesting a red algal-like photosynthetic endosymbiont [46], [47]. Thus, from an evolutionary viewpoint, the chloroplasts are fairly unique because they possess the remnants of a eukaryotic nucleus, the nucleomorph [48], [49], [50], [51]. Some genes may be encoded in the chloroplast or nucleomorph, which help the endosymbiont to adapt to the intracellular environment of the host. In addition, some alterations may have occurred in the genes or the ultrastructure of the chloroplasts, which help to integrate the newly engulfed organelles into the host cell. The nucleomorph appears to be important for the chloroplasts because it is retained in the host cell in most cases [20], [21], [22], [23], [24]. During the secondary endosymbiosis of cryptomonads, the genes encoded in the nucleomorph are highly compacted and most of the genes with metabolic functions are eliminated, leaving 30 genes for chloroplast-located proteins [52]. Gene transfer and reduction may occur once more in the nucleomorph after engulfment by the dinoflagellates. In a recent study of *Dinophysis acuminata*, horizontal gene transfer was detected from the kleptoplastidal chloroplast obtained from a cryptomonad to the host nucleus [21], which supports our hypothesis. In addition, endosymbiont genes were relocated to the nucleus via massive gene transfer in *Karenia brevis*, although the endosymbiont was a haptophyte instead of a cryptomonad, but the plastid still originated via a red algal secondary endosymbiosis [53]. The cryptomonad nucleus is probably not as important as the nucleomorph for the survival of the symbiont, because it is frequently lost in the host cell [20], [21], [24]. The lack of a cryptomonad nucleus in some dinoflagellates did not appear to affect the cell's ability to photosynthesize or move in response to varying levels of illumination [20]. In the present study, the genes encoded by the cryptomonad nucleus could not be amplified, although we tried many times, which suggested that the nucleus was probably lost.

All these unique characteristics might help the chloroplasts to adapt to new intracellular environments, although no clear evidence was available until now. Thus, it is necessary to study kleptoplastidal dinoflagellates and cryptomonads using comparative genomics and biochemistry methods to achieve a better understanding of the evolution of chloroplasts and algae.

## Materials and Methods

### Sample collection

Samples of *G. eucyaneum* were collected in April 2012 from Lake Donghu (30°32′55′′ N, 114°21′15′′ E) and from a fishpond in Wuhan, Hubei Province, China (30°28′34′′ N, 114°21′41′′ E) in May 2012, where it bloomed and accounted for more than 99% of the phytoplankton cell density. Samples of *Cryptomonas obovata, Cr. marssonii, Cr. ozolini, Cr. ovata, C. coerulea, Campylomonas reflexa*, and *Plagioselmis nanoplanctica* were collected from Lake Donghu in April 2012. Living cells were delivered immediately to the laboratory. Cells were kept cold during transportation. No specific permits were required for the described field studies. The locations where the samples collected from were not privately-owned or protected in any way, and the field studies did not involve endangered or protected species. Cells were isolated with the serial dilution pipetting technique [54] under an inverted microscope (CKX41; Olympus, Tokyo, Japan) and cultivated in sterilized lake water added with Bold's Basal Medium at a final concentration of 4%. Cells were grown at 17±2°C under a light: dark cycle of 14∶10 h (light intensity between 15 and 30 µmol photons m−2 s−1).

### Morphological observation

Living cells and cells fixed with Lugol's solution at a final concentration of 1% were observed using differential interference contrast (DIC), phase contrast (PH), and epifluorescence microscopy (EFM) with a light microscope (Leica DM5000B, Wetzlar, Germany). Micrographs were captured using a digital camera (Leica DFC320, Wetzlar, Germany).

### Spectrophotometry

Fishpond samples of *G. eucyaneum* and cultivated strain of *C. coerulea* were used. A 10 ml aliquot of the samples was pelleted for 3 min at 10,000×*g* and resuspended in phosphate buffer at pH 7.2 on three occasions. The cells were broken up by three cycles of freezing at −80°C and thawing. The cell debris was pelleted at 15,000×*g*. The absorption spectra of the supernatant was measured and recorded using a UV-1700 PharmaSpec spectrofluorometer (Shimadzu, Kyoto, Japan).

### DNA extraction, PCR amplification, and DNA sequencing

Approximately 50 *G. eucyaneum* cells were isolated using a serial dilution pipetting technique [54] under an inverted microscope (CKX41; Olympus, Tokyo, Japan). The cryptomonads were isolated in the same way and cultivated in sterilized lake water with 4% Bold's Basal (BB) Medium added [55]. The total DNA was extracted using the CTAB method [56].

The partial nuclear LSU rDNA sequence was amplified using the primers described by Scholin et al. [57]. The conditions used for PCR amplification of the partial LSU rDNA sequence and thermal cycling were those described in Hansen et al. [6]. The specific primers for the nucleomorph SSU rDNA (CMsF, 5′-TGGCT CATTA CAACA GTTAT AG-3′; CMsR, 5′-AGGCA TTCCT CGTTC AAG-3′) and chloroplast 23S rDNA (Cr23S1F, 5′-CAATA GATGC CTGTA CCTTA AACC-3′; Cr23S1R, 5′-TGGAC CGAAC TGTCT CACG-3′) of the endosymbionts were designed based on the conserved areas of known sequences of cryptomonads. The amplification profile of the nucleomorph SSU rDNA consisted of an initial denaturation step at 94°C for 4 min, followed by 35 cycles of 1 min at 94°C, 1 min at 60°C, and 90 s at 72°C, with 10 min at 72°C for the final extension. The chloroplast 23S rDNA PCR amplification started with 4 min at 94°C, followed by 35 cycles of 1 min at 94°C, 1 min at 55°C, 75 s at 72°C, ending with a final hold of 10 min at 72°C. All PCR amplicons were cleaned using an E.Z.N.A. Gel Extraction Kit (Omega Bio-Tek, Norcross, GA, USA), before some PCR products were cloned into the pMD18-T vector (Takara, Dalian, China). All clones were sequenced using the universal sequencing primer M13 with an ABI 3700 sequencer (Applied Biosystems, Foster City, CA, USA). The sequences were deposited in GenBank under the Accession Nos JX470945∼JX470953, JX545331, and JQ639750.

### Phylogenetic analyses

The sequences of putative relatives were downloaded from GenBank. Initially, the sequences were aligned using ClustalX 1.83 [58] and refined manually in SEAVIEW [59]. The final alignment of LSU rDNA sequences comprised a matrix of 38 sequences, including *Perkinsus marinus* (Perkinsozoa) as an outgroup taxon. A total of 32 cryptomonads, a dinoflagellate, and putative relative taxa were selected for the nucleomorph SSU rDNA sequence analyses. Species of Bangiophyceae were used as outgroup phylogenies because a previous study showed that members of the Bangiophyceae had close relationships with the nucleomorphs in cryptomonad cells [60]. The alignment of chloroplast 23S rDNA sequences comprised a matrix of 28 sequences including three blue-green algae as an outgroup.

Mutational saturation was evaluated in the variable positions of the alignments by plotting the pairwise distances against model-corrected distances for Tamura and Nei (1993) and Kimura (1980) models, which were estimated using MEGA (v.4.0) [61].

The phylogenies were estimated using the maximum-likelihood (ML) method in PAUP 4.0*(v. 4.0 beta) [62] and Bayesian inference (BI) in MrBayes (v. 3.1.2) [63]. For the ML analysis, ModelTest 3.06 [64] was used to select the evolutionarily best fit model given Akaike's Information Criterion (AIC). The best fit model for the LSU rDNA datasets was TrN+I+G. The best fit model for the nucleomorph SSU rDNA and chloroplast 23S rDNA datasets were GTR+I+G. In the ML analysis, a heuristic search option with random added sequences (100 replicates) and the tree bisection and reconnection branch-swapping algorithm were used for tree searching. A bootstrap analysis with 1000 replicates of the ML dataset was performed to estimate the statistical reliability. A Bayesian Markov Chain Monte Carlo analysis was conducted with seven Markov chains (six heated chains and one cold) for 5,000,000 generations, with the trees sampled every 1000 generations. The first 25% of the trees (burn-in samples) were discarded and the remaining samples were used to construct a Bayesian consensus tree and to infer the posterior probability.
